# Santa Claus in Great Ormond Street

**Published:** 1914-01-03

**Authors:** 


### Santa Claus in Great Ormond Street.

Christmas, essentially the children's festival, has, of course, a very special significance in a children's hospital, and nowhere, I think, can one better prove the truth of the words "that one finds one's own truest happiness in the happiness of others" than here. The sole thought in everybody's minds, from the matron down to the newest "pro.," is the happiness of the poor little sufferers, and for a short time at least pain and suffering take a back seat and happy smiling little faces greet one on every side. Excitement begins very early, for, of course, good Old Santa Claus has been busy during the night and a nice fat, bulgy stocking lies at the foot of each of the 260 small cots. The wards, with their red shades and screen covers, and red and white lights and flowers, present the most charmingly "Christmassy" appearance, and where everyone has taken so much trouble with the decorations it is hardly fair to criticise, but the very original lamp shades made to represent tomatoes which were hung in "Victoria" deserve a special word of praise, as also the tasteful appearance and general effect of "Alexandra" and "Annie Zunz." Dinner with the customary plum-pudding for those well enough to enjoy it is the next important event in this day of wonders, and great is the excitement when the pudding, holly decked and ablaze, is carried with all due ceremony into the ward. After dinner perhaps the greatest treat of all is in store, for then Santa Claus in person, real live Santa Claus in red gown and flowing beard, visits each ward and distributes to every little patient some much wanted toy! Nor is Santa Claus alone; with him, arrayed in fearful and wonderful disguise, are the resident medical staff, and loud is the laughter and great the excitement of children and nurses alike at their jokes and acrobatic achievements. Tea with a splendid iced cake and crackers in plenty is enjoyed in every ward, and many members of the visiting staff are to be seen making a tour of inspection, often stopping at one of the little cots to recognise or inquire after its small occupant. After tea Christmas carols are sung, and then the children, tired but happy, are quite ready to be settled off for the night. So ends what has been for some of the children the most wonderful as well as the happiest day they have ever known in their small lives, and to many of the nurses, tired though they may be with their efforts to amuse the little ones, still with the happy feeling that their efforts have indeed been amply repaid.

